# Community-Level Knowledge and Perceptions of Stroke in Rural Malawi: A Cross-Sectional, Population-Based Survey

**DOI:** 10.1161/STROKEAHA.119.025105

**Published:** 2019-06-05

**Authors:** Hazzie Mvula, Christina Chisambo, Vitumbiko Nyirenda, Steffen Geis, Judith R. Glynn, Amelia C. Crampin, Moffat Nyirenda, Liam Smeeth, Richard Walker, Alison J. Price

**Affiliations:** 1 From the Malawi Epidemiology and Intervention Research Unit, Lilongwe and Karonga (H.M., C.C., V.N., S.G., A.C.C., M.N., A.J.P.); 2 Faculty of Epidemiology and Population Health, London School of Hygiene and Tropical Medicine, United Kingdom (S.G., J.R.G., A.C.C., M.N., L.S., A.J.P.); 3 Northumbria Healthcare NHS Foundation Trust and Institute of Health and Society, Newcastle University, United Kingdom (R.W.).

**Keywords:** knowledge, Malawi, stroke, witchcraft

## Abstract

**Background and Purpose—:**

The incidence of stroke in Malawi is unknown but major risk factors, including hypertension, obesity, and diabetes mellitus, are highly prevalent. We sought to understand community-level knowledge about stroke.

**Methods—:**

A population-based cross-sectional study was conducted in rural Malawi (2016–2017). Adults aged ≥15 years were randomly selected and interviewed about their knowledge and perceptions of stroke symptoms, risk factors, and prevention. Logistic regression was used to investigate sociodemographic factors associated with stroke knowledge.

**Results—:**

Of 812 selected, 739 (91% response rate) were seen and consented; 57% were female, and the median age was 52.0 years. Knowledge of stroke was poor: 71% knew no (correct) risk factors. Witchcraft (20.6%) was mentioned as frequently as hypertension (19.8%) as a cause. Knowledge of stroke was greatest in the most educated and wealthy and lowest in men, the never married, and the youngest age group. HIV-positive individuals had higher knowledge of prevention (odds ratio, 2.91; 95% CI, 1.21–7.03) than HIV negative individuals.

**Conclusions—:**

Knowledge about stroke is very low in this community, particularly among the least educated and poor. Programs to support prevention, early recognition, and timely hospital presentation after a stroke are needed.

Stroke is a major cause of morbidity and mortality in adults worldwide^1^; 80% of stroke-related disability-adjusted-life-years and 75% of deaths occur in low- and middle-income countries.^2^

Population-based stroke studies in sub-Saharan Africa are few,^3^ yet stroke incidence in some urban and middle-income African populations exceeds that observed in developed countries.^4^ Cardiovascular risk factors, such as hypertension, obesity, and diabetes mellitus, are also common^5^ (even in very poor countries like Malawi) and largely uncontrolled^6^; rendering individuals vulnerable to stroke and other cardiovascular diseases within constrained health systems.

Effective stroke prevention and management require a good understanding of the condition. In Africa^7^ and elsewhere,^8^ poor knowledge of stroke symptoms and risk factors have been shown to delay health care seeking after a stroke. Sociocultural beliefs and the use of traditional healers may also lead to delay in accessing care and interruption of treatment.^9^

We explored knowledge of stroke in a rural Malawian community to provide baseline information for planning stroke prevention and management interventions.

## Methods

Our study was conducted in the rural Karonga Health and Demographic Surveillance Site, northern Malawi (n=42 000).^6^ We used our population database as the sampling frame and stratified random sampling, within 10-year age bands, and 21 geographic areas, to select a sample size of 812 adult residents (15+ years); 80% power to detect an odds ratio of 1.6 with alpha set at 0.05 for a 2-sided hypothesis test. Eligible individuals were “missing” if they were not found within 3 household visits.

The data that support the findings of this study are available from the corresponding author on reasonable request. We used interviewer-led open-ended questions, comparable to data from published studies,^10–14^ related to stroke signs and symptoms, risk factors, prevention measures, and actions to help a person with stroke. The questionnaire was translated into the local language (Chitumbuka) and back-translated by experienced local translators and piloted. The English word “stroke” was used throughout as it is widely used, and there is no direct Chitumbuka translation. The term “kufwa viwalo” translating directly as “limb paralysis” is inappropriate as it includes other causes of paralysis, such as polio. Written consent and enrollment were conducted from June 2016 to March 2017.

Participants were encouraged to list as many responses as possible for each question without prompting. Clarifications to questions were provided where necessary. Completed questionnaire responses were scored against a list of acceptable responses by 2 independent clinical staff, with double entry, verification, and error correction. Knowledge was categorized a priori as good (≥5 correct responses), fair (2–4 correct) and poor (0–1 correct).

We used logistic regression models (STATA.V-12.Stata-corp, TX) to investigate factors associated with knowledge of stroke symptoms, risk factors, and prevention measures: participant’s age, sex, education, marital status, and socioeconomic status. HIV status and distance to the nearest health facility were also explored because of potential impact on access to health services and messages. Final models included adjustment for factors significant in bivariate analyses at *P*<0.20.

Ethical approval was obtained from the Malawi National Health Science Research Committee; protocol No. 1324.

## Results

Of 812 randomly selected individuals, 742 (91.4%) were seen, and 739 (91%) consented and interviewed. Educational achievement, distance to the nearest health center, and sex were similar for eligible participants (n=739) and nonparticipants (n=73; *P*>0.05), but nonparticipants were younger, poorer, and more likely to be never married (*P*<0.001; results not shown). Participant median age was 52.0 years (interquartile range, 34.1–69.7).

Very few participants had good knowledge (≥5 correct responses) of stroke symptoms (n=25; 3.4%), risk factors (n=2; 0.3%), or prevention measures (n=2; 0.3%). Most study participants did not know any stroke risk factor (n=525; 71.0%) or prevention measures (564; 76.3%) but were able to identify at least 1 correct sign or symptom (529; 71.6%; Table 1). Hypertension was the most common correct risk factor response (n=146; 19.8%) and witchcraft (n=152; 20.6%) the most common incorrect response. HIV infection was not mentioned as a stroke risk factor by anyone.

**Table 1.**
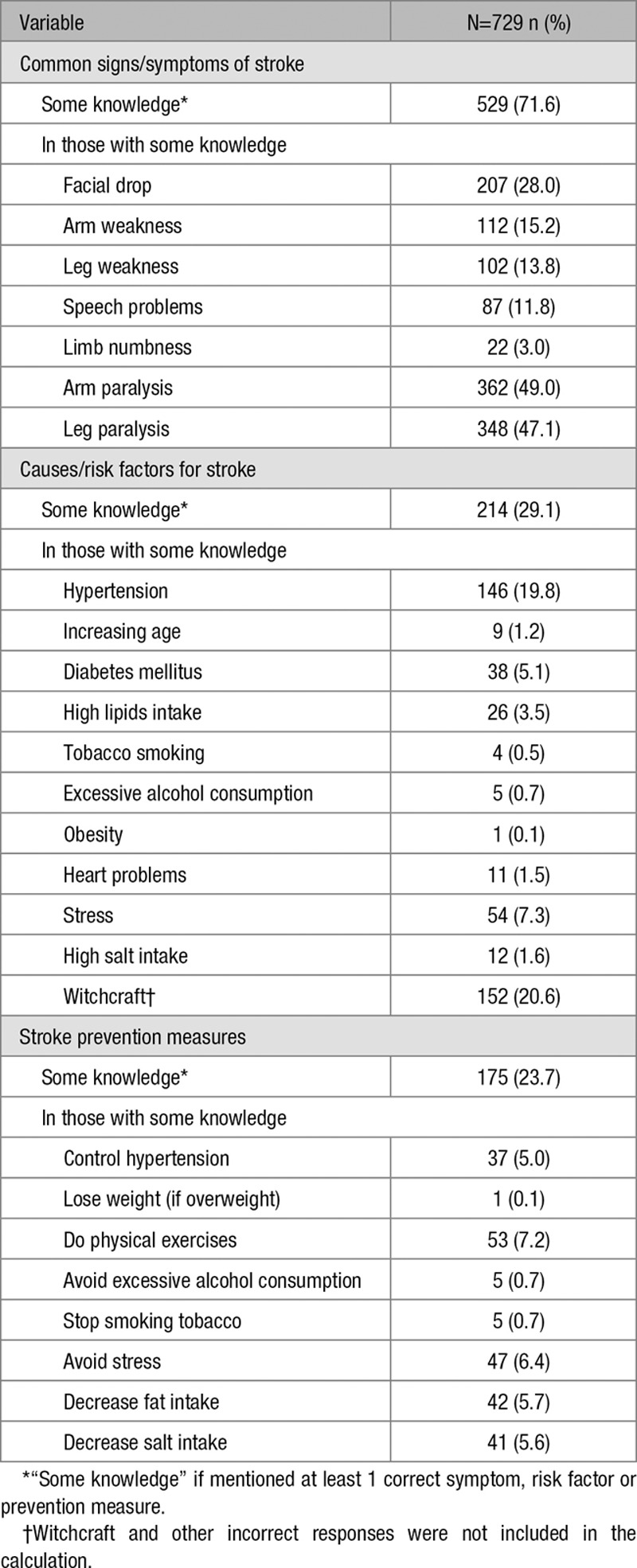
Stroke Symptoms, Risk Factors and Prevention Measures Mentioned by Participants

Knowledge was low hence further analyses compared at least 1 (correct) response versus none for each outcome (Table 2). The groups with the least knowledge of stroke symptoms, risk factors, and prevention were the least educated, the poorest, men, the never married, and those living close to a health center. Those known to be HIV positive had greater knowledge of stroke prevention measures.

**Table 2.**
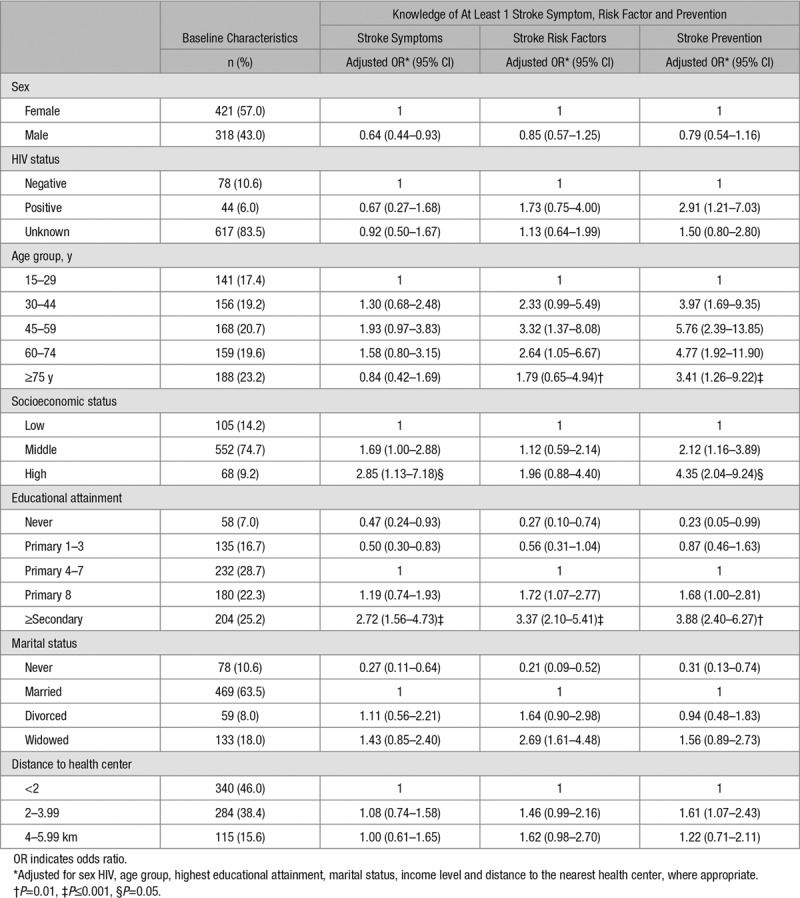
Odds Ratios of the Association Between Baseline Characteristics and Stroke Knowledge

There is no emergency response call-service in Malawi. Most respondents (n=596; 80.7%) reported that they would take a relative or friend to hospital for acute stroke, but few (n=50; 6.8%) knew of any treatment (results not shown).

## Discussion

In this population, knowledge of stroke symptoms, risk factors, and prevention measures was very limited. Although most people (71.7%) correctly described at least 1 stroke symptom, few (<30%) were able to correctly list a single stroke risk factor or prevention measure.

Low knowledge has been found elsewhere. Paralysis symptoms were described by less than half of our study participants; comparable to findings from Ghana (38.0%), Benin (34.4%), and Uganda (28.6%).^11,13,14^

Although adult hypertension prevalence is high,^11^ few participants (19.8%) mentioned hypertension as a risk factor; lower than observed in Nigeria (88.6%) and Uganda (56%)^12,13^ and perhaps related to low levels of hypertension diagnosis and treatment in Malawi.^11^

Witchcraft was the most frequently proposed risk factor (20.6%) in our study, comparable to findings from hospital workers in Nigeria (13.8%)^12^ and Ghana (26%)^14^ but higher than urban and rural Uganda (0.9%)^13^ and urban Benin (4.3%).^11^ The extent to which this belief affects the health care seeking behavior of those who experience a stroke, warrants further investigation.^13^

Although the prevalence of HIV in this community is around 8%, HIV was not perceived as a stroke risk factor. The higher knowledge of prevention in HIV-positive participants may reflect greater access to health messages for those in HIV care. Greater stroke knowledge in those of higher education and socioeconomic status has been reported elsewhere.^12,13^ This may, in part, be because of the higher prevalence of hypertension and diabetes mellitus and associated care in these groups.^6^ In our setting, the middle age groups were the most knowledgeable. A recent systematic review reported inconsistent findings in the association between knowledge of stroke warning signs and age.^10^

Our study was conducted in a community setting where participants were selected at random within age and geographic strata. Given our high response rate and an age and sex structure similar to national rural population estimates,^6^ our findings should be generalizable to other rural settings in Malawi where 80% of the population reside. Despite the high prevalence of cardiometabolic risk factors,^6^ there is poor understanding of the associated long-term-health trajectories and missed opportunities for primary prevention and effective, timely treatment. We have previously shown that only 60% of individuals screened for hypertension in the community took up clinic referral, and of those started on treatment for hypertension, <50% were in care at 1 year.^15^ A limited understanding of cardiovascular disease risk may be an important contributing factor.

## Conclusions

In rural Malawi, knowledge of stroke symptoms, risk factors, and preventative measures is very low, particularly among the poorest and least educated, and misperceptions that witchcraft plays a role persist. Educational interventions that reach those with poor knowledge, at high risk (the elderly and those with cardiometabolic disease), and care-providers are needed to prevent and manage a likely growing burden of stroke in this population.

## Sources of Funding

Supported by Wellcome postdoctoral fellowship funding (A.J. Price).

## Disclosures

None.

## Supplementary Material

SUPPLEMENTARY MATERIAL
