# Supplementary material for: Community-Level Knowledge and Perceptions of Stroke in Rural Malawi: A Cross-Sectional, Population-Based Survey
Source: Stroke. 2019 Jun 5;50(7):1846–9. doi: 10.1161/STROKEAHA.119.025105 (PMC6594749; doi:10.1161/STROKEAHA.119.025105)
Supplement: SUPPLEMENTARY MATERIAL [file str-50-1846-s001.pdf]

SUPPLEMENTAL MATERIAL

Supplemental Table I

RECOVER Trial intervention components checklist

Item No.	Items
**Brief name**	
**1**	Stroke rehabilitation intervention for disabled population in rural China
**Why**	
**2**	To deliver the stroke rehabilitation skills to the patients with stroke in low-resource contexts, the task-shifting model was used to transfer the responsibility of rehabilitation physicians and therapists to nurses in county hospitals and then the trained nurses delivered skills to caregivers and/or patients before discharge as well as provided caregivers and/or patients with follow-up training via three telephone calls after discharge. Caregivers delivered these exercises to patients and supported them to practice every day at home post-discharge.
**What**	
**3**	Materials: 1) a picture-rich and easy-to-understand intervention manual; 2) a self-videotaped intervention guidance VCD; 3) in-hospital intervention implementation flowchart; 4) the rehabilitation planning form; and 5) an android system-based app.
**4**	Procedures: 1) Psychological assessment and cooperative assessment; 2) Assessment of physical function, daily reminders and five exercise principles; 3) Filling in the nurse-used training plan form; 4) The requirement of in-hospital training; 5) Implementation of the training

	in -hospital; 6) Instruction of pre-discharge; 7) Three telephone follow-ups.
**Who provided**	
**5**	1) Training-the-trainer (nurses): Rehabilitation physicians and therapists provided nurses with rehabilitation skills centrally;<br>2) Training the caregivers and/or patients: Nurses provided caregivers and/or patients with rehabilitation skills in-hospital;<br>3) Delivering intervention to patients: Caregivers delivered skills to patients and supported them at home.
**How**	
**6**	1) Nurses were trained by accredited rehabilitation physicians and therapists at Peking University First Hospital centrally;<br>2) Nurses provided caregivers and/or patients with training in-hospital, including designing individualized training plan for patients, face-to-face training to caregivers and patients, providing them with instruction of pre-discharge, and also providing them with three follow-ups after discharge;<br>3) Caregivers delivered these exercises to patients and supported them to practice every day at home post-discharge.
**Where**	
**7**	In-hospital and patients' homes
**When and how much**	
**8**	1) A whole training session would last approximately 90 minutes during three to six times and be conducted over a period of 2-3 days;

	2) Nurses provided three telephone follow-up support to caregivers at weeks 2, 4 and 8.
**Tailoring**	
**9**	The physical function rehabilitation section is individualized and focused on five rehabilitation aspects measured by the three BI domains and four key movements in the assessment tools. For example, if a patient was not able to make his bed or chair move, he needed the training of item 3 and 5 in the chapter of free movement in the intervention manual.
**Modifications**	
**10**	No any modifications
**How well**	
**11**	Planned: A team will conduct process evaluation to assess the fidelity of intervention implementation.
**12**	Actual: Basically, nurses provided intervention following protocol to caregivers, but we have no opportunities to observe how caregivers delivered training to patients after discharge.

Supplemental Table II. The results of qualitative review

Roles	Quotes
**Nurses**	**Some positive feedback:**<br>• All nurses reported that the intervention manual and the RECOVER app was useful tool;<br>– *When we input some personal information to RECOVER app, this app can tell us which rehabilitation skills should be trained to patients and their caregivers, and what the page number of rehab manual we can find these skills in. It takes about one and a half hours delivering these necessary rehabilitation skills to them, according to the guidance of rehabilitation manual which is a kind of picture-rich manual and very easy to follow. In a word, we believe we have trained them with all skills that they need to study. – A nurse from Zhangwu County Hospital*<br>– *This is a practical and user-friendly app, and helps me a lot when I implement intervention and enter data. It can always remind me what the next step is and which rehabilitation skills I should deliver to patients and their caregivers. – A nurse from Qingtongxia County Hospital*<br>• All nurses were very interested in taking part in this trial and showed their inspiration on participating in future stroke rehabilitation studies;<br>– *This is a really interesting and meaningful study, contributing a lot to stroke patients and their family caregivers in rural China, because our hospital involves some basic nursing work in our routine, but never a systematic rehabilitation program on stroke patients and we learn a lot from this study this time as well as hope to participate in future projects*

related to stroke rehabilitation. – A nurse from Dianjiang County Hospital

Some negative feedback (barriers):

- Six out of seven nurses reported that the rehabilitation skills were a little complex to them and not very easy to deliver;
  - *I did not study any stroke rehabilitation before, so I feel the current skills are still a little complex to us, although we have received training three times. I hope to simplify more rehabilitation skills, so that I could master easily and manage patients. – A nurse from Qingtongxia County Hospital*
- Five out of seven nurses thought that the study imposed extra workload on them;
  - *Actually, I have a stressful workload on my routine work, and in most cases, I have to spend my extra time completing the training tasks, even in my holidays. – A nurse from Zhangwu County Hospital*
- Two out of seven nurses reflected that the network disconnected sometimes when using the *app*;
  - *Sometimes I cannot log in my account. Maybe network has some problems, and I have to wait until it runs well. – A Nurse from Zhangwu County Hospital*
- All nurses found that more than one caregivers taking turns to take care of patients made complete caregiver skill training difficult;
  - *Around one third patients have above one family caregivers, who are*

	more likely to take turns caring for patients, so that in this case, other caregivers will take care when patients discharged instead of the caregiver I have trained in hospital. Furthermore, those untrained caregivers are not willing to be trained once patients discharged, and I have no time to visit their home to train them again. – A nurse from Dianjiang County Hospital
**Patients /caregivers**	• All patients / caregivers were able to follow these exercises but may not be so “smooth”;<br>– *Nurses are very nice to me, and they taught me how to exercise and pose my limbs, but sometimes a part of skills I cannot be very easy to imitate smoothly. In a word, I hope to recover soon, and I am willing to follow nurses’ guide and exercise myself. – a patient from Qingtongxia*<br>• Four out of six caregivers / patients were unable to deliver or exercise after discharge as well as they did in the hospital.<br>– *My caregiver is my son. He is always busy and seldom goes home. I know I should have exercised my leg every day, but I have no courage to practice without my son, and I hope the nurse who trained me in county hospital can help me but I know she is also very busy. – a patient from Zhangwu*
